# Periorbital hyperpigmentation as the initial manifestation of smoldering multiple myeloma in a patient with coexisting gastrointestinal stromal tumor: a case report and narrative literature review

**DOI:** 10.3389/fonc.2026.1908738

**Published:** 2026-09-14

**Authors:** Yuze Yang, Wenyan Xu, Xiangli Li, Zhenxing Guo

**Affiliations:** 1 Department of Hematology/Oncology, The First Hospital of Tsinghua University, School of Clinical Medicine, Tsinghua Medicine, Tsinghua University, Beijing, China; 2 Department of Pathology, The First Hospital of Tsinghua University, School of Clinical Medicine, Tsinghua Medicine, Tsinghua University, Beijing, China

**Keywords:** case report, dual primary malignancies, gastrointestinal stromal tumor, periorbital hyperpigmentation, smoldering multiple myeloma

## Abstract

Periorbital hyperpigmentation (POH) as the sole initial manifestation remains undescribed in patients with smoldering multiple myeloma (SMM) without amyloid deposition, and the coexistence of SMM with gastrointestinal stromal tumor (GIST) is exceptionally rare. We describe a 56-year-old male who was treated with imatinib for eight months after resection of a low-risk gastric GIST and developed bilateral symmetric POH. Workup revealed IgA κ-type monoclonal gammopathy and 3% abnormal plasma cells on bone marrow aspiration. Further biopsies of bone marrow and abdominal wall fat excluded amyloid deposition. Therefore, a diagnosis of SMM (IgA κ-type) was made. To the best of our knowledge, this is the first reported case of SMM presenting with isolated POH in the absence of light-chain (AL) amyloidosis and coexisting with a GIST. This case suggests that POH may represent a rare early cutaneous manifestation of SMM and may further broaden the recognized clinical spectrum of SMM.

## Introduction

Multiple myeloma (MM) is a hematologic malignancy characterized by clonal proliferation of abnormal plasma cells in the bone marrow, secretion of monoclonal immunoglobulin, and subsequent organ dysfunction. Smoldering multiple myeloma (SMM), a subtype of asymptomatic myeloma, typically lacks immediate treatment indications and is managed primarily with regular follow-up (1). Gastrointestinal stromal tumor (GIST) is the most common mesenchymal neoplasm of the gastrointestinal tract. Patients with low-risk GIST achieve a favorable prognosis after surgery combined with targeted therapy, with imatinib serving as the first-line adjuvant targeted therapy (2). To our knowledge, metachronous dual primary tumors of SMM and GIST are rarely reported, and periorbital hyperpigmentation (POH) as the initial manifestation of SMM is even rarer. This article reports a case of SMM initially presenting as isolated POH in a patient with a coexisting GIST. We also review the relevant literature to enhance our understanding of the disease and to provide useful information for clinical diagnosis.

## Case presentation

A 56-year-old male patient presented to the Department of Gastroenterology of our hospital on November 18, 2024, with intermittent hematochezia for 6 days. Contrast-enhanced whole-abdominal CT revealed a soft tissue density lesion adjacent to the left lateral segment of the liver, approximately 2.8 × 1.8 cm, with ill-defined borders between the lesion, liver tissue, and gastric wall, of undetermined nature (**Figure 1A**). Contrast-enhanced upper abdominal MRI showed an abnormally enhancing lesion adjacent to the left lateral segment of the liver, measuring approximately 3.0 × 2.0 cm, suggestive of a hepatic lesion, possibly a hemangioma (**Figure 1B**). Gastroscopy demonstrated an extrinsic compression lesion on the anterior wall of the lower gastric body near the greater curvature, measuring 3.0 × 3.0 cm, with a smooth surface and firm consistency on palpation with forceps. On December 11, 2024, the patient underwent laparoscopic partial gastrectomy and lysis of intestinal adhesions. Intraoperative exploration revealed significant adhesions in the left upper abdominal region. Further adhesiolysis confirmed that the mass originated from the stomach. Finally, intraoperative pathological diagnosis was a low-risk GIST originating from the gastric wall (**Figure 2A**). Immunohistochemistry (IHC) staining results were as follows (**Figures 2B–F**): DOG1 (++), VEGF (focal +), CD117 (++), CD34 (+), Ki-67 (hotspot area ~5%), SMA (-), S-100 (-). From December 27, 2024, the patient received imatinib 400 mg once daily as adjuvant therapy and was followed up regularly without recurrence. On July 14, 2025, the patient presented to the Outpatient Clinic of Hematology and Oncology with a 2-month history of periorbital darkening and denied skin purpura, edema, ecchymosis, macroglossia, or bone pain. Serum immunofixation electrophoresis (IFE) was positive for IgA κ. The patient had a past medical history of hypertension, coronary heart disease, and chronic hepatitis B virus infection, with no history of radiation exposure or family history of tumors.

**Figure 1:**
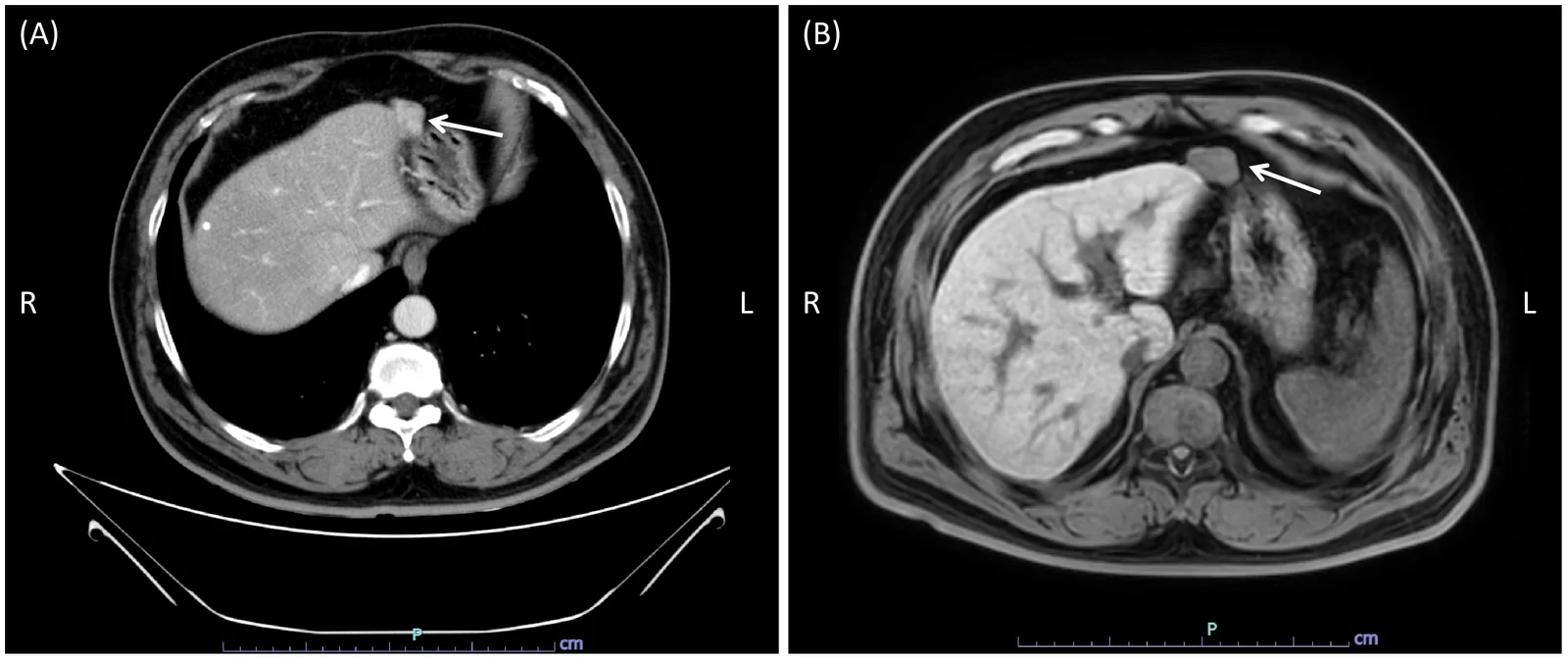
Abdominal imaging of the patient. **(A)** Contrast-enhanced whole-abdominal CT (axial view) shows a soft tissue density lesion adjacent to the left lateral segment of the liver (arrow). **(B)** Contrast-enhanced MRI of the liver, gallbladder, pancreas, and spleen (axial view) shows an abnormally enhancing lesion adjacent to the left lateral segment of the liver (arrow).

**Figure 2:**
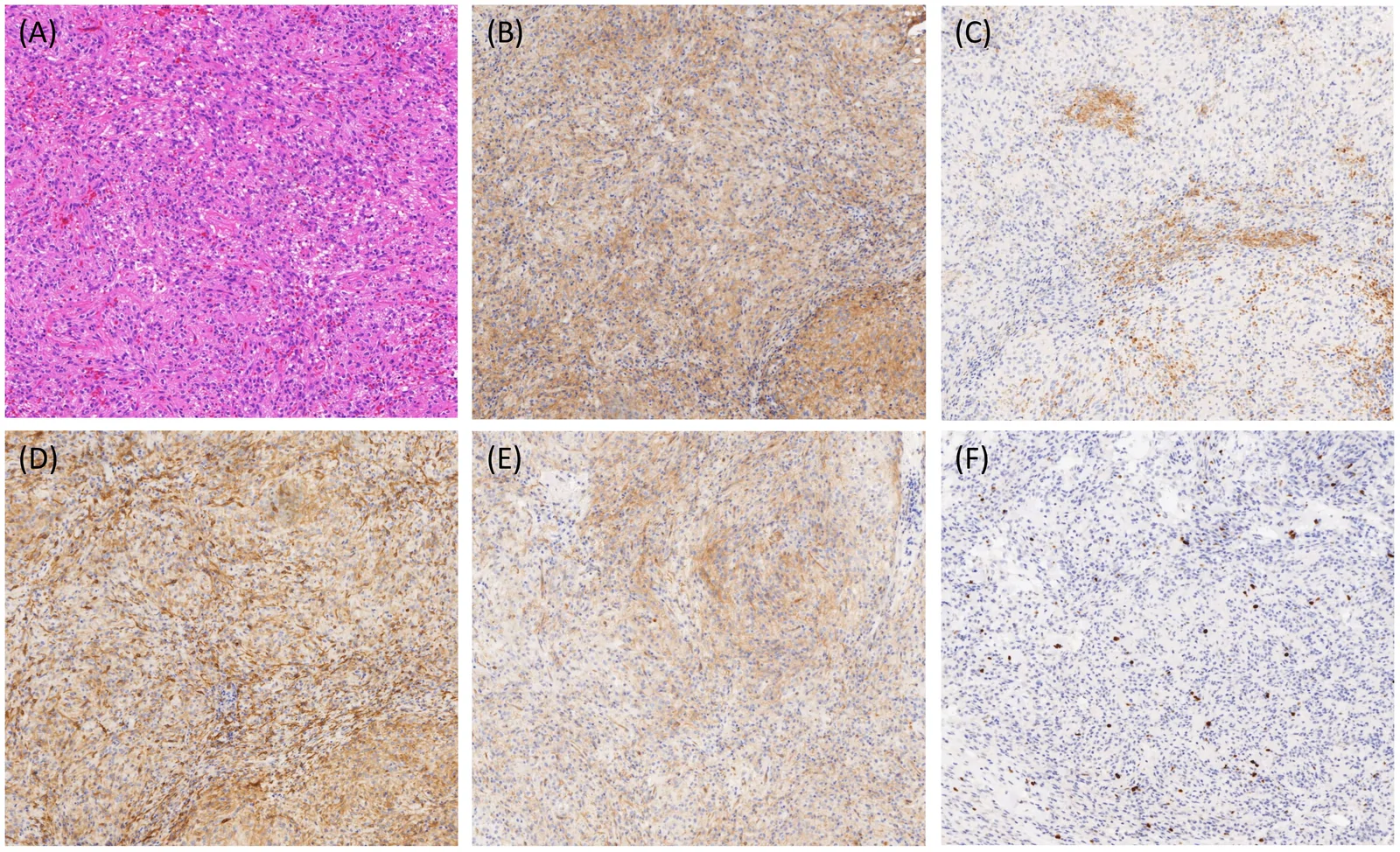
Pathological findings of the gastric GIST (×400). **(A)** HE staining: spindle cell tumor. **(B)** DOG1: (++). **(C)** VEGF: focal (+). **(D)** CD117: (++). **(E)** CD34: (+). **(F)** Ki-67: ~5% (hotspot). The tumor was negative for SMA and S-100 (data not shown).

Physical examination revealed the patient had bilateral symmetric, diffuse grayish-brown POH (**Figure 3**). No scleral icterus or lip pallor was observed. There were no palpable enlarged lymph nodes or sternal tenderness. Cardiopulmonary and abdominal examinations were unremarkable. Further laboratory tests showed the following: IgA 10.45 g/L (reference range 1.0–4.2 g/L, ↑); IgG 4.6 g/L (reference range 8.6–17.4 g/L, ↓); IgM <0.2 g/L (reference range 0.3–2.2 g/L, ↓); serum κ light chain 3.43 g/L (reference range 1.38–3.75 g/L); serum λ light chain <0.5 g/L (reference range 0.93–2.42 g/L, ↓). Serum IFE was positive for IgA κ-type M protein, with an M protein quantification of 10.45 g/L and a κ/λ light chain ratio of 6.96 (reference range 1.15–2.95, ↑). Urine IFE was positive for κ-type light chain M protein, with urine κ light chain of 128.99 mg/L (reference range <10 mg/L, ↑) and a urine κ/λ ratio of 23.45 (reference range 0.72–4.6, ↑). All other tests, including complete blood count, coagulation profile, liver/kidney function, electrolytes, myocardial enzymes, and autoantibodies, were within normal limits. Bone marrow morphology revealed hypocellular marrow with abnormal plasma cells accounting for 3%. Flow cytometry analysis showed that CD38^+^/CD138^+^ cells constituted 1.6% of total cells; these cells expressed CD56 and CD200 but lacked CD19, CD27, CD81, CD20, CD28, CD34, CD117, and CD33. Cytoplasmic Ig light chain was restricted to κ, consistent with an abnormal plasma cell phenotype. Chromosome karyotyping revealed no abnormalities. Fluorescence *in situ* hybridization (FISH) of bone marrow was negative for probes targeting 1p32, 1q21, D13S319/LAMP1, RB1, IGH, and p53/CEP17. Bone marrow biopsy (**Figure 4**) showed hypocellular marrow with plasma cell myeloma. CD138^+^ cells accounted for approximately 10% of nucleated cells and were restricted to κ light chain. Congo red staining was negative. Abdominal wall fat pad biopsy with colloidal gold immunoelectron microscopy labeling revealed no amyloid deposition. According to the International Myeloma Working Group (IMWG) criteria, the diagnosis of SMM requires ≥10% clonal bone marrow plasma cells and/or serum M protein ≥30 g/L, in the absence of end-organ damage (3). In this case, bone marrow biopsy showed approximately 10% CD138-positive plasma cells with κ light chain restriction and an IgA κ-type M protein of 10.45 g/L. Importantly, the patient had no CRAB features (hypercalcemia, renal impairment, anemia, or bone lesions). Furthermore, positron emission tomography-computed tomography (PET-CT) demonstrated postoperative changes of GIST without extramedullary lesions. Whole-spine MRI revealed no vertebral infiltration. Therefore, the patient was diagnosed with low-risk SMM (IgA κ-type) coexisting with a low-risk gastric GIST (modified NIH criteria) (4, 5).

**Figure 3:**
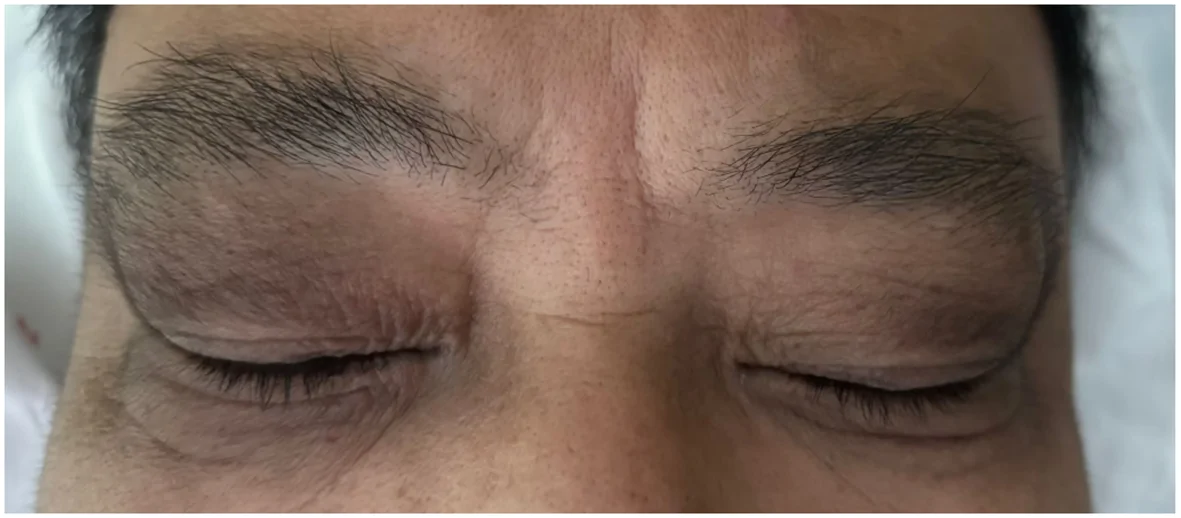
Periorbital hyperpigmentation in the patient (eyes closed). Bilateral symmetric, diffuse grayish-brown hyperpigmentation is observed around the orbits.

**Figure 4:**
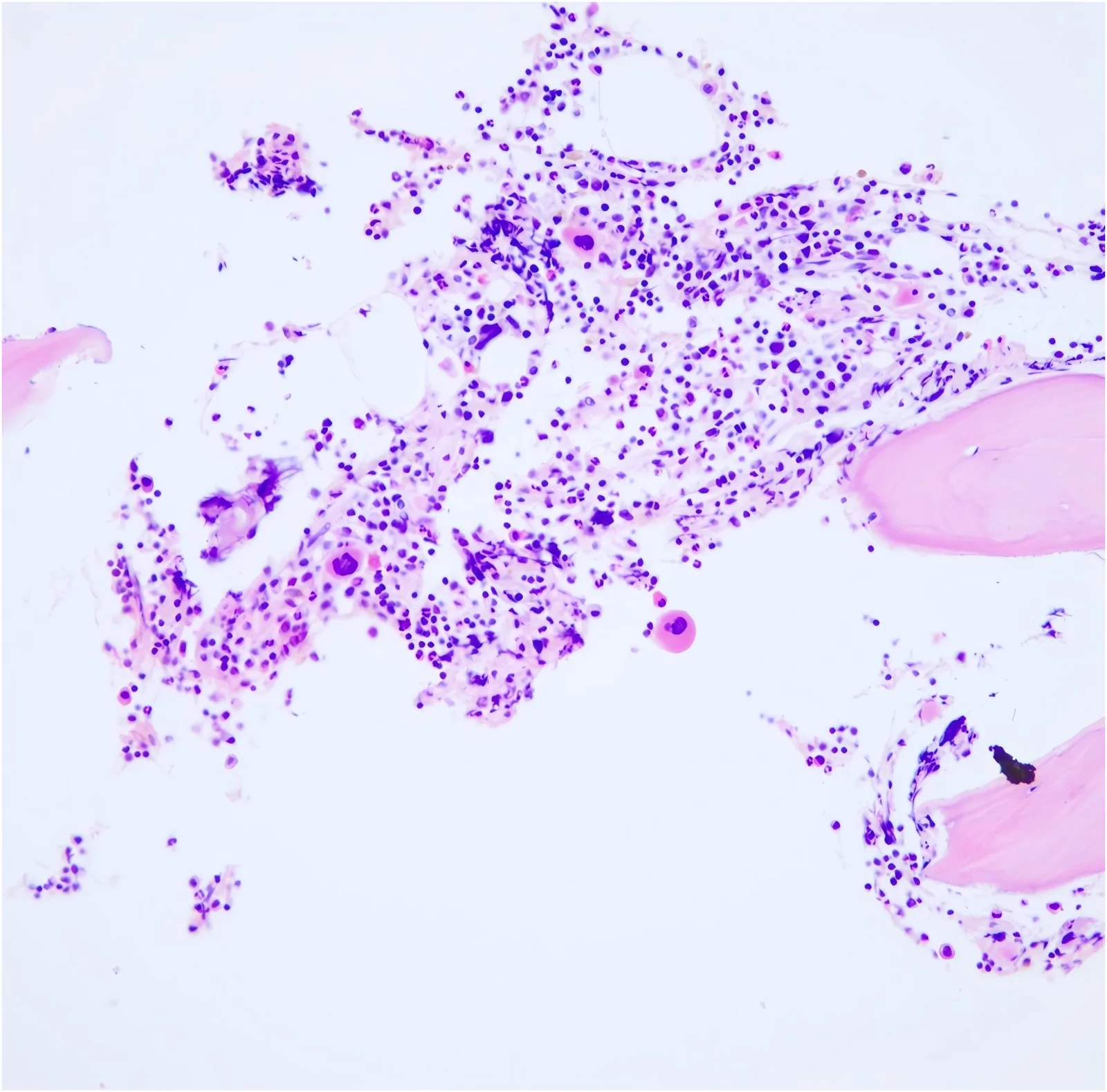
Pathological findings of bone marrow biopsy (HE, ×200). The bone marrow is hypocellular, with abnormal plasma cells distributed in small patches and scattered foci.

## Patient perspective

The patient acknowledged being informed about the rarity of his symptom and the importance of long-term hematological surveillance. He reported good tolerance to imatinib and confirmed his willingness to continue regular follow-up.

## Discussion

We report a rare case of SMM presenting with isolated POH as the sole initial manifestation, in the absence of light-chain (AL) amyloidosis, and coexisting with a GIST. This unusual presentation posed a diagnostic challenge, as acquired POH has a broad differential diagnosis. Our patient had bilateral symmetric POH without pruritus or edema. The pigmentation emerged after imatinib initiation, and concurrent monoclonal gammopathy was detected during the same period. Therefore, we performed a differential diagnostic evaluation to exclude AL amyloidosis, POEMS syndrome, drug-induced pigmentation, and other potential etiologies.

Periorbital ecchymosis, the classic ‘raccoon eyes’ sign, is a characteristic cutaneous manifestation of AL amyloidosis, typically attributed to amyloid deposition in periorbital vessel walls or acquired factor X deficiency (6). The present case clinically resembled this sign. AL amyloidosis is relatively common in MM (7, 8), and this association reinforced our diagnostic consideration, with one prospective study reporting that 18.7% of patients with systemic AL amyloidosis met the diagnostic criteria for MM (9). However, in our patient, neither Congo red staining of the bone marrow biopsy nor immunoelectron microscopy of the abdominal wall fat pad biopsy revealed amyloid deposits, thereby ruling out AL amyloidosis as the underlying cause of the POH. Additionally, POEMS syndrome required further diagnostic consideration. The diagnosis of POEMS requires the presence of both mandatory criteria (polyneuropathy and a monoclonal plasma cell disorder), plus at least one major criterion and one minor criterion (10). In our patient, the absence of polyneuropathy, organomegaly, and endocrinopathy effectively excluded this diagnosis. Other acquired causes of POH, such as Addison’s disease or post-inflammatory hyperpigmentation, were excluded, as the patient had no clinical or laboratory evidence of these conditions.

Given the onset of POH approximately eight months after imatinib initiation, we needed to consider drug‑induced pigmentation as a potential cause. Imatinib is well known to cause cutaneous pigmentary changes, most commonly hypopigmentation resulting from inhibition of c‑kit‑mediated melanogenesis (11), although hyperpigmentation has also been reported (12, 13). However, imatinib‑associated hyperpigmentation predominantly involves photoexposed facial areas such as the forehead and malar regions, with eyelids typically spared (13, 14). To the best of our knowledge, it has not been documented as isolated POH. Additionally, imatinib‑induced hyperpigmentation typically emerges within 2 to 6 months of treatment initiation (13, 14), whereas the pigmentation in our patient appeared at 8 months, a less typical timeline. Furthermore, the POH was observed concurrently with an IgA κ-type monoclonal gammopathy and clonal plasma cells in the bone marrow, a temporal association that cannot be readily explained by drug exposure alone. Of note, the patient remained on imatinib with no progressive worsening of the POH. Therefore, while a contributory role of imatinib cannot be definitively excluded, these observations suggest that SMM represents a more plausible explanation for the POH.

To contextualize our findings, we conducted a focused narrative literature review. We searched PubMed up to June 2026 using keywords including “periorbital hyperpigmentation,” “plasma cell disorders,” “multiple myeloma,” “amyloidosis,” and “cutaneous manifestation.” Reference lists of retrieved articles were also manually screened. Based on this search, we summarized previously reported cases of unusual periorbital cutaneous manifestations associated with plasma cell disorders in **Table 1**.

**Table 1:** Summary of unusual periorbital cutaneous manifestations associated with plasma cell disorders in the literature.

Author, year	Underlying diagnosis	periorbital cutaneous manifestations	Amyloidosis	Ref.
Rappaport K et al., 1999	Primary extramedullary plasmacytoma	Periorbital swelling/edema/erythema (recurrent cellulitis) with orbital hematic cyst	Not reported	(19)
Vilcassim FS, et al., 2017	Active MM with AL amyloidosis	Coalescing pigmented periorbital papules	Present	(20)
Kikuchi N et al., 2019	Active MM with AL amyloidosis	Xanthoma-like yellowish plaques with petechiae	Present	(21)
Koutra E, et al., 2025	Active MM	Diffuse plane xanthomas (yellow macules/patches in periorbital region)	Not reported	(22)
Lee SH, et al., 2019	Active MM	Diffuse normolipemic plane xanthomatosis (DNPX) with periorbital yellowish plaques	Present	(23)
Cheema HI, et al., 2026	SMM	Ulcerative periorbital lesions (NXG) with orbital mass effect	Excluded	(24)
Bouattour N, et al., 2020	SMM	Periorbital swelling with mild erythema (secondary to superior ophthalmic vein thrombosis)	Not reported	(25)
Yang YZ, et al., 2026	SMM	Isolated bilateral periorbital hyperpigmentation	Excluded	—

Active MM, active multiple myeloma; AL, amyloid light-chain; DNPX, diffuse normolipemic plane xanthomatosis; SMM, smoldering multiple myeloma; NXG, necrobiotic xanthogranuloma.

As summarized in **Table 1**, the periorbital cutaneous manifestations reported in plasma cell disorders are diverse, most of which are associated with AL amyloidosis. In contrast, our patient presented with isolated, bilateral symmetric diffuse grayish‑brown hyperpigmentation without evidence of amyloid deposition. This pattern differs from previously reported xanthomatous plaques, swelling/edema, and nodular or ulcerative lesions and, to the best of our knowledge, has not been described in SMM. This observation suggests that POH may represent a rare and early cutaneous manifestation of SMM.

SMM and GIST are pathogenetically distinct, originating from plasma cells and interstitial cells of Cajal, respectively (1, 15). Their coexistence constitutes a rare form of multiple primary malignancies (MPMs) (16). Reports of metachronous SMM and GIST are extremely limited. Tzilves et al. previously reported a case of a patient who developed symptomatic MM after 14 months of GIST resection (17). Similarly, our patient exhibited a temporal pattern with an interval of approximately 8 months between the two diagnoses, meeting the definition of metachronous MPMs (16). Up to now, the pathogenesis of MPMs remains poorly understood, with proposed contributing factors including host susceptibility, treatment-related effects and environmental exposures (18). However, whether the coexistence of SMM and GIST in our patient represents a causal link or a coincidental event remains uncertain and cannot be determined from a single case observation.

This case suggests that POH may represent a rare early cutaneous manifestation of SMM, and may further broaden the recognized clinical spectrum of SMM. The temporal sequence of the two malignancies in our patient suggests that metachronous tumors may occur in succession and that their clinical manifestations may mask each other. Therefore, a multidisciplinary approach may be emphasized in the management of such patients. During long‑term follow‑up of patients with known malignancies, comprehensive systemic evaluation may be prioritized over focusing solely on the initial tumor. However, several limitations of the present case should be acknowledged. This is only a single-center, single-case observation, which precludes causality or generalizability. The potential contribution of imatinib to POH cannot be definitively excluded, and long‑term follow‑up data are insufficient. Further accumulation of cases is warranted to better define the clinical significance of these findings.

## Conclusion

In summary, we report a rare case of SMM presenting with isolated POH as the initial manifestation in the absence of AL amyloidosis, coexisting with a GIST. This case suggests that isolated POH may represent a rare and early cutaneous manifestation of SMM. The patient’s long‑term prognosis warrants continued follow‑up, and more cases are needed to clarify the clinical significance of this finding.

## Data Availability

The original contributions presented in the study are included in the article/supplementary material. Further inquiries can be directed to the corresponding author.
